# Expression of HLA-DR by mesenchymal stromal cells in the platelet lysate era: an obsolete release criterion for MSCs?

**DOI:** 10.1186/s12967-023-04684-5

**Published:** 2024-01-09

**Authors:** Zyrafete Kuçi, Natascha Piede, Kathrin Vogelsang, Lisa-Marie Pfeffermann, Sibylle Wehner, Emilia Salzmann-Manrique, Miriam Stais, Hermann Kreyenberg, Halvard Bonig, Peter Bader, Selim Kuçi

**Affiliations:** 1 Department for Children and Adolescents, Division for Stem Cell Transplantation, Immunology and Intensive Care Medicine, Goethe University Frankfurt, University Hospital, Theodor-Stern-Kai 7, 60590 Frankfurt am Main, Germany; 2 Institute for Transfusion Medicine and Immunohematology, Goethe University and German Red Cross Blood Service BaWüHe, Institute Frankfurt, Frankfurt, Germany

**Keywords:** Mesenchymal stromal cells, Platelet lysates, Fetal bovine serum, HLA-DR expression, MSC-release criteria

## Abstract

**Background:**

According to the definition of the International Society for Cell and Gene Therapy (ISCT), mesenchymal stromal cells (MSCs) do not express HLA-DR. This phenotypic marker as a release criterion for clinical use was established at a time when MSCs were expanded in fetal bovine serum (FBS)-containing media. Replacement of FBS with platelet lysate (PLs) as a medium supplement induced a significantly higher fraction of MSCs to express MHC class II antigens.

**Methods:**

As this raised concerns that such MSCs may play the role of antigen-presenting cells for T cells, in the current study, we studied major factors that may induce HLA-DR on MSCs by means of flow cytometry and real-time polymerase chain reaction. The immunomodulatory potential of MSCs was assessed by a mixed lymphocyte reaction.

**Results:**

Our results demonstrated that a very low percentage of generated and expanded MSCs in FBS express HLA-DR (median: 1.1%, range: 0.3–22%) compared to MSCs generated and expanded in PLs (median: 28.4%, range: 3.3–73.7%). Analysis of the cytokine composition of ten PLs showed a significant positive correlation between the levels of IL-1β, IL-4, IL-10, IL-17, bFGF and expression of HLA-DR, in contrast to no correlation with the age of MSC donors and HLA-DR (r = 0.21). Both MSCs expressing low and high levels of HLA-DR expressed class II transactivator (CIITA), a master gene coding for these molecules. Our results demonstrate for the first time that MSCs with constitutively high levels of HLA-DR also express moderate levels of indoleamine 2,3-dioxygenase (IDO). Treatment of MSCs with multiple doses of TGF-β1 at passage 0 (P0) and passage 1 (P1) completely abrogated HLA-DR and IDO expression. In contrast, treatment of MSCs with a single dose of TGF-β1 after P0 only partially reduced the expression of HLA-DR and CIITA. Remarkably, increased expression of HLA-DR on MSCs that constitutively express high levels of this antigen after overnight incubation with IFN-γ was rather unaffected by incubation with TGF-β1. However, treatment of MSCs with TGF-β1 for 24 h completely abrogated constitutive expression of IDO.

**Conclusions:**

Irrespective of HLA-DR expression at the population level, all MSC preparations significantly inhibited the proliferation of stimulated peripheral blood mononuclear cells, indicating that HLA-DR represents an obsolete release marker for the clinical use of MSCs.

## Introduction

Mesenchymal stromal cells (MSCs), as nonhematopoietic cells, are widely used in the clinic because they can be easily generated and expanded ex vivo. MSCs are able to differentiate into various tissues and may be used in both autologous and allogeneic clinical settings. Because of their potential to suppress immune responses in vitro [1–6], they represent an attractive source of cells for in vivo modulation of excessive immune responses in the clinic [7–10] as well as in regenerative medicine [11–14].

According to the definition of the International Society for Cell and Gene Therapy (ISCT) [15], MSCs should fulfill some minimal criteria, e.g., MSCs should have fibroblast-like morphology, grow as plastic-adherent cells and have the capacity to differentiate into three lineages (osteoblasts, chondrocytes and adipocytes). They should also express a specific phenotype, such as CD73, CD90, CD105, and CD44, and should be negative for hematopoietic cell surface molecules, such as CD45, CD34, CD14/CD11b, CD19/CD79α and CD31. Moreover, they should not express HLA-DR (MHC class II), which plays a crucial role in antigen presentation to T cells and therefore leads to their activation and differentiation. The transcriptional activation of class II MHC genes requires the induction of the class II transactivator (CIITA) protein**,** a master regulator that is essential for both constitutive and IFN-γ-inducible class II MHC expression [16–18].

Tang et al. [19] demonstrated that nontreated MSCs generated from unfractionated bone marrow aspirates with FBS as a medium supplement express minute amounts of CIITA in comparison to IFN-γ-stimulated MSCs. In contrast, Bocelli-Tyndall et al. [20] reported that nontreated MSCs do not express CIITA unless they were treated with bFGF. According to these authors, TGF-β1 could abrogate CIITA expression completely. Similarly, the cytokine IL-1β has been demonstrated to inhibit IFN-γ-induced CIITA gene expression [16].

Thus far, in the majority of studies, MSCs have been generated and expanded in media supplemented with fetal bovine serum (FBS) as a source of growth factors. Therefore, there was a common consensus that MSCs expanded in FBS do not constitutively express HLA-DR. However, it has been also shown that irrespective of their expansion in FBS-containing media, a higher percentage of MSCs expressed of HLA-DR [21]. It then occurred when the MSCs were generated from complete bone marrow and not from the isolated bone marrow mononuclear cell (BM-MNC) fraction [22].

When treated with IFN-γ, the majority of MSCs express HLA-DR [2–4, 23–26]. Increased expression of this molecule may also be induced by culture of MSCs in the presence of bFGF, a cytokine usually used to improve the proliferation rate of MSCs [20, 22, 27]. TGF-β1 exerts an inhibitory effect on the expression of HLA-DR on MSCs induced either by IFN-γ, [25], or bFGF [20]. IL-1β is another molecule that decreases the expression of HLA-DR after the treatment of MSCs with bFGF [28].

In addition to HLA-DR, T-cell costimulatory molecules such as CD80 (B7-1), CD86 (B7-2) and CD40 play an important role in the induction of the immune response. Similar to HLA-DR, costimulatory molecules are not constitutively expressed [3, 29–31]. Their expression by MSCs does not increase even when the expression of HLA-DR increases after stimulation with IFN-γ or bFGF [4, 20, 32]. As MSCs do not express costimulatory molecules, they cannot *bona fide* serve as antigen-presenting cells and therefore may not be immunogenic. In line with this, even MSCs that express high levels of HLA-DR after IFN-γ stimulation or bFGF treatment in a mixed lymphocyte reaction (MLR) with peripheral blood mononuclear cells (PB-MNCs) from one allogeneic donor were not able to induce the proliferation of allogeneic mononuclear cells [4, 22, 23, 27, 32].

In contrast, MSCs actively suppressed the proliferation of responder PB-MNCs stimulated by third-party allogeneic PB-MNCs as well as T cells stimulated by anti-CD3 and anti-CD28 antibodies [4], as has also been demonstrated in many other reports [2, 22, 27, 32].

In the current study, we investigated various components that may affect the HLA-DR marker as a release criterion of MSC preparations for clinical use: donors of the bone marrow, the supplement for the basal culture medium (FBS vs. PL), including differences between batches of the supplements and their cytokine composition.

## Material and methods

### Collection of platelet concentrates and generation of PLs

As a starting material for PLs, we used pooled platelets from 1- to 2-day-old buffy coats from whole blood containing approximately 10% platelet additive solution III.

To generate platelet lysate, four platelet concentrates containing platelets from four donors each were pooled as one batch, aliquoted in sterile 50 mL Falcon tubes and immediately frozen at − 80 °C. Individual aliquots were thawed after at least 24 h and centrifuged for 20 min at 3500*g*. The supernatants (PLs) were collected and used to generate and expand MSCs. In total, 36 batches of PLs were used in the current study. In 16 PLs, the cytokine profile was determined, and 10 of these 16 PLs were used to generate MSCs. Thirty-five PL batches were used for determination of bFGF concentrations with ELISA.

### Bone marrow samples

Bone marrow aspirates were obtained from the posterior iliac crest of healthy donors (9–47 years old) following informed consent, according to guidelines approved by the local Ethics Committee of German Red Cross Blood Donor Service Baden-Württemberg/Hessen, Frankfurt, Germany (vote #352/10).

BM-MNCs were isolated by density gradient centrifugation (Pancoll human, Cat. No P04601000, Pan-Biotech, Aidenbach, Germany).

To analyze factors that can influence HLA-DR expression, in the current study, we generated MSCs from the bone marrow of 33 donors. Seventeen donors were males, 16 were females, and 15/33 donors were under 20 years old. MSCs from 23 donors were generated by using “MSC-qualified” FBS (Gibco/Invitrogen, Cat. No 12662–029, Darmstadt, Germany). For the generation and expansion of MSCs in PLs, 15 batches of PLs were used.

### Generation of MSCs

One volume of the bone marrow was diluted at a 1:3 ratio with PBS and after mixing was layered on a Ficoll gradient. After centrifugation for 25 min at 700*g,* mononuclear cells were isolated and counted. To generate MSCs, BM-MNCs were plated in culture flasks at a density of 1.72 × 10^5^ cells/cm^2^ in DMEM (Dulbecco’s modified Eagle’s medium low glucose, Gibco/Invitrogen, Cat. No 31966–021, Darmstadt, Germany) containing 10% “MSC-qualified” FBS or 5% PL and 5 IU heparin/mL DMEM. Cells were incubated in an incubator with 5% CO_2_ and 95% humidity at 37 °C. After 72 h, the medium containing nonadherent cells was removed and replaced with fresh medium. The adherent cells were cultured for an additional 13 days until reaching approximately 80–90% confluence. During this time, the medium was changed every 3 days. Primary MSCs were detached using TrypLE (Gibco, Cat. No 12563011 Darmstadt, Germany) and thereafter were seeded at a density of 2 × 10^3^ MSCs/cm^2^ with DMEM containing 10% PL and 5 IU heparin/mL DMEM or 10% FBS. At the end of the cell culture, primary MSCs (P0) were further expanded for another passage (P1). MSCs of both passages were used for determination of HLA-DR expression.

### Determination of the phenotype of MSCs

The MSC phenotype was evaluated by immunostaining MSCs with the following fluorochrome-conjugated antibodies: IgG1-FITC (Cat. No 400110, Clone MOPC-21), IgG2a-FITC (Cat. No 400210, Clone MOPC-173), IgG1-PE (Cat. No 400114, Clone MOPC-21), IgG1-APC (Cat. No 400122, Clone MOPC-21), and HLA-DR-FITC (Cat. No 307604, Clone L243). The L243 monoclonal antibody reacts with the HLA-DR antigen, a member of MHC class II molecules, and does not cross react with HLA-DP and HLA-DQ.), CD80-PE (Cat. No 305208, Clone 2D10), CD86-APC (Cat. No 374208, Clone BU63), CD40-PE, (Cat. No 334308, Clone 5C3), CD90-FITC, (Cat. No 328108, Clone 5E10) or CD90-APC/Fire 750 (Cat. No 328138, Clone 5E10) CD73-FITC (Cat. No 344016, Clone AD2), CD105-PE, (Cat. No 323206, Clone 43A3), CD19-PerCP/Cy5.5 (Cat. No 302230, Clone HIB19), CD14 BV421 (Cat. No 325628, Clone HCD14), CD45 BV786 (Cat. No 304048, Clone HI30), CD34 Alexa Fluor 700 (Cat. No 343526, Clone 581) anti-HLA-DR BV605 (Cat. No 307640, Clone L243).

All antibodies and the appropriate conjugated isotype controls were purchased from BioLegend (Koblenz, Germany).

### Isolation of PB-MNCs

PB-MNCs were isolated from buffy coats of healthy adult volunteers collected by the German Red Cross Blood Donor Service Baden-Württemberg/Hessen, Frankfurt, with written informed consent and permission of the local Ethics Committee (vote #329/10). PB-MNCs were isolated by density gradient after overlaying on a Ficoll gradient (Pancoll human).

To track PB-MNC proliferation using flow cytometry, isolated PB-MNCs from freshly prepared buffy coats were stained with Violet Proliferation Dye 450 (VPD450 BD, Cat. No 562158 BD Horizon, Heidelberg, Germany) (3 µM f.c.). After 10 min of incubation in a water bath in the dark at 50 rpm, stained PB-MNCs were washed with PBS, followed by a subsequent washing step with RPMI 1640 (Roswell Park Memorial Institute 1640 Medium, Gibco, Cat. No 61870010, Darmstadt, Germany) supplemented with 10% FBS. The number of cells was adjusted to 2 × 10^6^ PB-MNCs/mL in RPMI-1640 supplemented with 10% FBS and thereafter used in the MLR.

### Mixed lymphocyte reaction (MLR)

#### Immunosuppressive potential of MSCs

To study the immunosuppressive effect of MSCs, they were cocultured with VPD450-stained freshly isolated PB-MNCs that were stimulated with 0.4 μg/ml anti-human CD3 and CD28 antibodies (Cat. No 300332, Clone HIT3a, and Cat. No 302934, Clone CD28.2, respectively; Biolegend, Koblenz, Germany).

A total of 6 × 10^5^ PB-MNCs/600 µL RPMI supplemented with 10% FBS were incubated with or without 6 × 10^5^ third-party lethally irradiated (30 Gy) MSCs in 300 µL RPMI/10% FBS per well in a 24-well plate. PB-MNCs in designated wells were stimulated with 300 µL anti-CD3/anti-CD28 antibodies (1.6 µg/mL)/RPMI supplemented with 10% FBS at a final concentration of 0.4 µg/mL.

The cells were incubated for 5 days in an incubator at 37 °C with 95% humidity and 5% CO_2_. On day 5, the cells were harvested from wells, and after washing twice with PBS, they were stained for flow cytometry measurement using a BD FACSLyric flow cytometer and FACSuite software 1.4 (BD Biosciences, Heidelberg, Germany).

### Immunogenicity

To test immunogenicity, 6 × 10^5^ lethally irradiated MSCs in 600 µL of RPMI supplemented with 10% FBS were cocultured with 6 × 10^5^ PB-MNCs/300 µL of RPMI supplemented with 10% FBS in the designated wells. In these wells, 300 µL of RPMI or 300 µL of RPMI with 1.8 µg/mL (f.c. 0.4 µg/mL) anti-CD28 antibodies was pipetted into 24-well plates. The cells were cocultured for 5 days in an incubator at 37 °C with 95% humidity and 5% CO_2_. On day 5, the cells were harvested, and after washing twice with PBS, they were stained for flow cytometry measurement. The following fluorochrome-conjugated monoclonal antibodies were used for determination of immune cell subsets: CD3-BV510 (Cat. No 300448, Clone UCHT1), CD4-Alexa Fluor 700 (Cat. No 344622, Clone SK3), CD8-Alexa Fluor 488 (Cat. No 344716, Clone SK1), CD45-BV786 (Cat. No 304048, Clone HI30) and the viability dye Zombie NIR (Cat. No 423106). All antibodies were purchased from BioLegend (Koblenz, Germany).

### Quantification of cytokine levels in PLs and FBS

To determine the levels of different cytokines in PLs and FBS that might play a role in the expression of HLA-DR by MSCs, we analyzed 5 batches of FBS and 16 batches of PLs for the following cytokines: IL-1β, IL-4, IL-10, IL-17, IFN-γ, IP-10 (CXCL10), PDGF-BB, TNF-α, TGF-β and bFGF. All these cytokines were measured by using a multiplex bead-based assay. In addition, a separate evaluation of bFGF in 35 different PL batches was performed by ELISA according to the manufacturer’s instructions (human FGF basic/FGF2, Quantikine Elisa, Cat. No DFB50, R&D Systems, Wiesbaden, Germany) by using a microplate reader, Victor^**③**^multilabel reader and Wallac 1420 Manager Software version 3.0 (Perkin Elmer, Rodgau, Germany).

### Real-time polymerase chain reaction (RT‒PCR)

To demonstrate the role of PLs and TGF-β1 in HLA-DR, IDO and CIITA expression, we selected MSCs from one donor that expressed high percentages of HLA-DR. MSCs from this donor were generated in 4 different batches of PLs. MSCs during generation were intermittently treated with TGF-β1 (10 ng/mL, 5 ng/mL and 2.5 ng/mL, Peprotech, Hamburg, Germany) on days 0, 3, 7 and 10. After trypsinization on day 13, during further P1, MSCs were treated on days 1 and 3. Depending on the proliferation rate, MSCs were trypsinized on day 6 or 7 and frozen for RNA isolation. Untreated MSCs were used as a control. To assess the effect of the treatment of MSCs with only one dose of TGF-β1 on the expression of HLA-DR, MSCs of P2 generated from 3 donors (2 in the same, one in a different PL) were treated with TGF-β1 alone (10 ng/mL), IFN-γ alone (500 IU/mL) and a combination of both (TGF-β1 + IFN-γ) during the last 24 h before trypsinization. Thereafter, the cell pellet was used for RT‒PCR. RNA was isolated by a NucleoSpinRNA plus Kit (MACHEREY–NAGEL, Cat. No 740984.50, Düren, Germany).

One to two micrograms RNA/20 µL was transcribed to cDNA with the High-Capacity cDNA Reverse Transcription Kit (Applied Biosystems, ThermoFisher Scientific, Cat. No 4368814, Darmstadt, Germany). For amplification of the samples, GoTaq Green Master Mix (Promega, Cat. No M7122, Walldorf, Germany) was used. For this purpose, 50 ng cDNA probe/25 µL PCR were used.

To determine the PCR product size and relative quantity of IDO, TGF-β1, CIITA upstream region and CIITA downstream region, as well as GAPDH as a housekeeping primer [33], the Agilent 2100 Bioanalyzer equipped with the 2100 Expert Software and the DNA 1000 LabChip Kit (Agilent Technologies, Palo Alto, CA, USA) was used for the detection of fragments ranging from 25 to 1000 bp. The RT‒PCR product of CIITA in the results is presented as the sum of the CIITA upstream and downstream regions (Table 1).

**Table 1:** Primers used for the amplification reaction

Target	Primer sequence	Product size (bp)	(References)
IDO	F 5‘-CGC TGT TGG AAA TAG CTT C-3’	234	[40]
	R 5‘-CAG GAC GTC AAA GCA CTG AA-3’		
TGF-ß1	F 5‘-CAG ATC CTC TCC AAG CTG-3’	270	[40]
	R 5‘-TCG GAG CTC TGA TGT GTT-3’		
CIITA- P/S/T	F-5‘-GAA AAG ACC CTT CCC AGAGG-3’	201	[19]
	R-5‘-GGG AAT CTG GTC GGT TTT CT-3’		
CIITA-LRR	F- 5‘-GGG AAA GCT TGT GCA GAC TC-3’	199	[19]
	R- 5‘-GGG AAA GCT TGT GCA GAC TC-3’		
GAPDH	F-5‘-CAC CAC CAT GGA GAA GGC TGG-3’	552	[33]
	R-5‘-GAA GTC AGA GGA GAC CAC CTG-3’		

PCR conditions used: 3 min at 95 °C/1 min at 96 °C; 3 temperatures for 10 cycles: 94 °C for 30 s, 64 °C (with a 1 °C decrease per cycle) for 30 s and 70 °C for 45 s; 3 temperatures for 28 cycles: 90 °C for 30 s, 55 °C for 30 s and 70 °C for 30 s; and hold at 60 °C for 30 min. RT‒PCR was analyzed with a capillary electrophoresis machine (2100 Bioanalyzer, Agilent, Germany)

## Statistical analysis

Statistical analyses were performed using GraphPad Prism 9.5.0 software (GraphPad Software, San Diego, CA, USA) and the software for statistical computing and graphics R version 4.3.0. All tests were two-tailed, and a *p* value of less than 0.05 was considered to be statistically significant. Normally distributed continuous variables are presented as the mean and standard deviation. Paired or unpaired t tests were used for their comparison, as appropriate. Unknown distributions of continuous variables are presented as medians and ranges, and we used paired or unpaired Wilcoxon tests, as appropriate, for their comparison. Spearman rank correlation coefficients (r) were calculated to evaluate the significance of soluble cytokine levels in PLs and HLA-DR expression in MSCs. As some of the above subgroups had small numbers, comparisons were performed by ad hoc analyses.

## Results and discussion

**Fig. 1:**
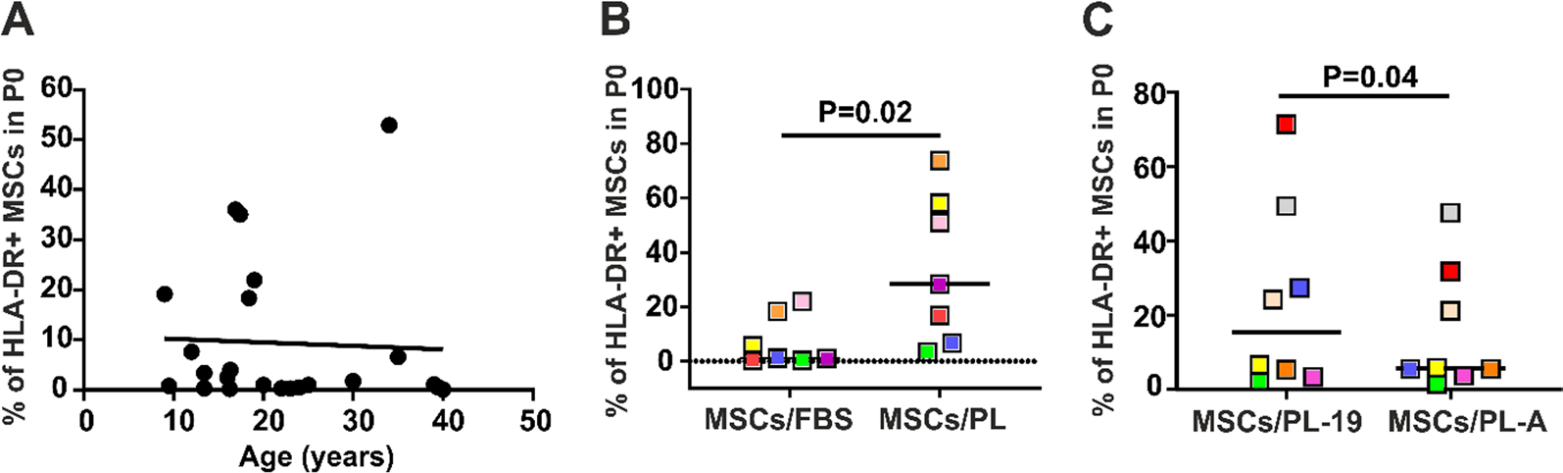
Donor, supplement of the cell culture medium and PL-batch affect HLA-DR expression by primary MSCs. **A** HLA-DR expression by MSCs, which were generated from the bone marrow of 23 donors, in DMEM supplemented with 10% FBS for 13 days (P0). Spearman correlation analysis showed no correlation between the age of MSC donors and HLA-DR expression (r = 0.21). **B** Primary MSCs (P0) were generated from bone marrow mononuclear cells of seven donors after thirteen days in culture with DMEM supplemented either with FBS (10%) or PL (5%). The median percentage of HLA-DR + MSCs/FBS was 1.1% (range 0.29–22%), whereas that of MSCs in PL was 28.4% (range 3.34–73.7%). The difference between the groups was statistically significant (*p* < 0.02; Wilcoxon signed-rank test). **C** Primary MSCs (P0) were generated from bone marrow mononuclear cells of eight donors after thirteen days in culture with DMEM supplemented with 5% of two different PLs: PL-19 and PL-A. The median percentage of HLA-DR + MSCs in PL-19 was 15.43% (range 2.7–71.4%), and that in PL-A was 5.6% (range 1.5–47.5%). The difference between the groups was statistically significant (*p* < 0.04; Wilcoxon signed-rank test). PL-19 and PL-A are labels for different PL batches. In panels B and C, each donor is represented with one colored square

In the current study, we investigated major factors that may affect HLA-DR expression by MSCs after their expansion in cell medium supplemented with PL or FBS. We classified these factors into 2 categories: donor-dependent and supplement dependent. In Fig. 1A, the percentage of primary MSCs (P0) expressing HLA-DR, which were generated from 23 donors in DMEM supplemented with 10% MSC-qualified FBS, is presented. Primary MSCs (P0) from 12 out of 23 donors (53.8%) expressed lower levels of HLA-DR than 2%, while the MSCs from 11 donors expressed higher levels of HLA-DR (up to 52.9%). In this group, 26.9% of MSCs expressed higher levels than 10% HLA-DR. No correlation between age and HLA-DR expression levels was observed.

Figure 1B shows the percentage of MSCs expressing HLA-DR antigen, which were generated from BM-MNCs of 7 donors in two different supplements: FBS and PL. A significantly higher percentage of MSCs expanded in PL expressed HLA-DR antigens compared to MSCs expanded in FBS (p < 0.02), as already observed in earlier reports [34]. However, the expression level of HLA-DR in MSCs from each donor expanded with PL did not correlate with the expression level of HLA-DR in MSCs expanded with FBS, indicating that in addition to the supplement, the donor-specific components also play a role in the expression of HLA-DR antigen. We also observed that MSCs of some donors expressed high levels of HLA-DR despite their culture in medium supplemented with FBS, in line with other reports [20, 28].

To assess the influence of the PL batch on HLA-DR expression, primary MSCs (P0) were generated from BM-MNCs of eight donors with 2 different batches of 5% PLs (PL-19 vs. PL-A) for thirteen days (Fig. 1C). The results showed that when a low percentage of primary MSCs from one donor constitutively express HLA-DR, in the majority of cases, platelet lysate does not strongly affect its expression. In contrast, if a higher percentage of MSCs from one donor express HLA-DR, then platelet lysate can significantly affect the increase or decrease in its expression.

**Fig. 2:**
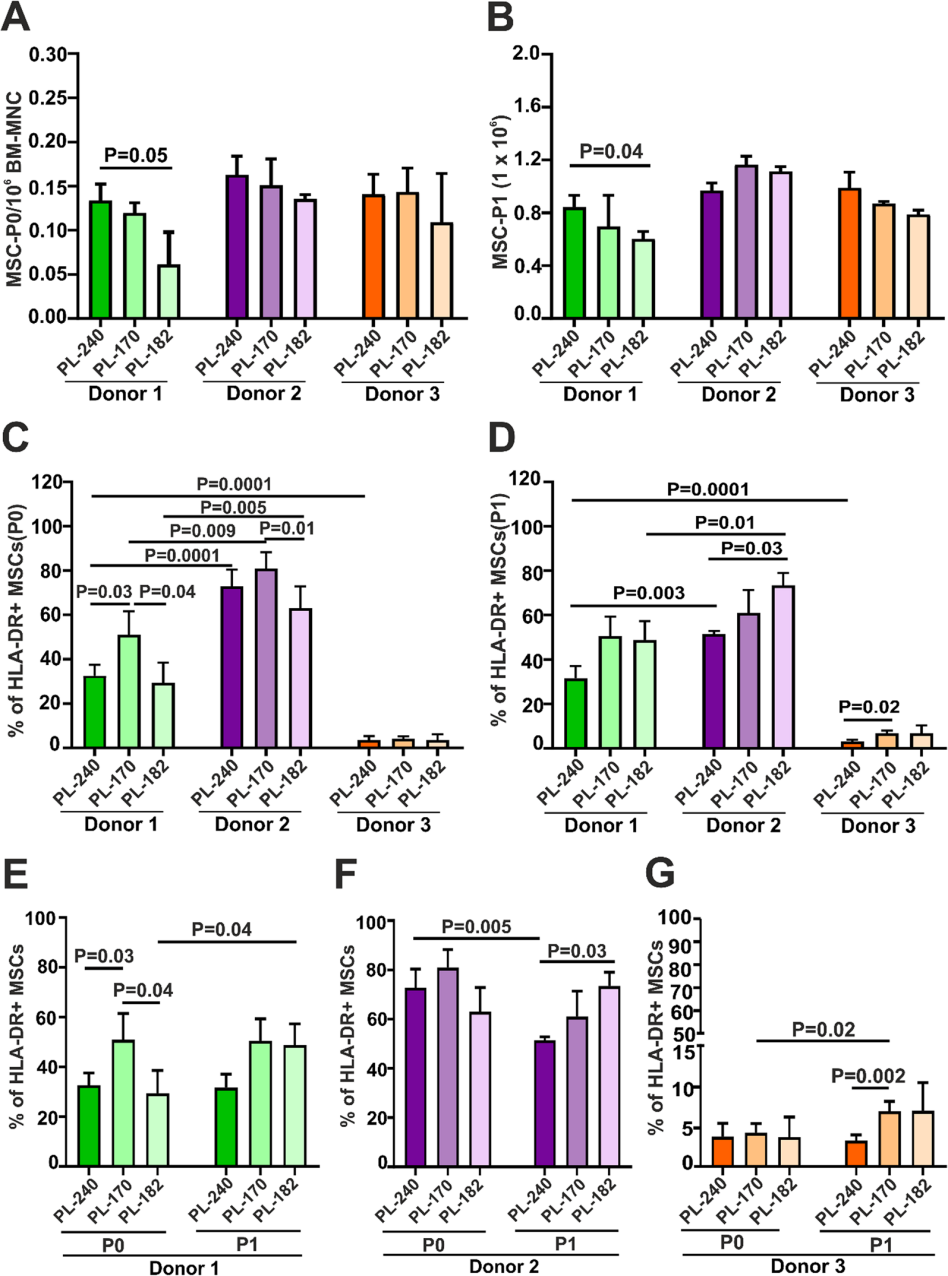
Expression of HLA-DR in primary MSCs (P0) and P1 generated from 3 different bone marrow donors in 3 different PLs. **A** Generation of MSCs from BM-MNCs. **B** Expansion of MSCs in 3 different PLs. Number of expanded MSC-P1 from 5 × 10^**5**^ MSC-P0. **C** Comparison of HLA-DR expression by MSCs of 3 donors in P0 and the same expanded MSCs in 3 different PLs in P1 (**D**). Comparison of HLA-DR expression by MSCs in P0 and P1 of donor 1 (**E**), donor 2 (**F**) and donor 3 (**G**). The results are presented as the mean ± SD of 3 experiments. Mean values from the same donor in different PLs were compared by using a paired t test, whereas comparison of mean values between different donors was performed by an unpaired t test

To further analyze the impact of donor, batches of PL and proliferative rate of MSCs on the expression of HLA-DR as well as its influence on the immunosuppressive effect in the MLR, we generated MSCs from 3 bone marrow donors, each with 3 different PLs within 13 days (Fig. 2A). Furthermore, the MSCs were expanded with the same PL batches but at a concentration of 10% PL for a week as P1 (Fig. 2B). MSCs from all three donors showed no significantly different proliferation potential at P0 and P1 (Fig. 2A, B). However, MSCs from donor 1 proliferated better in PL-240 than in PL-182 at P0 and P1 (*p* < 0.05, *p* < 0.04, respectively), but this difference in proliferation potential (Fig. 2A, B) did not affect HLA-DR expression (Fig. 2C).

We observed that MSCs from some donors genuinely expressed higher levels of HLA-DR (Fig. 2C: donors 1 and 2) than MSCs from other donors, whose MSCs expressed very low levels of HLA-DR (Fig. 2C: donor 3). Platelet lysate can also induce significantly different levels of expression of HLA-DR by P0 MSCs, as observed in donor 1 and donor 2 (Fig. 2C, D) and donor 3 (Fig. 2D). However, there was no correlation observed between the proliferation rate and expression levels of HLA-DR by primary MSCs (P0) or MSCs-P1. The most remarkable example of the lack of this correlation is donor 3, whose MSCs were generated very successfully in all 3 platelet lysates (Fig. 2A), but they expressed extremely low levels of HLA-DR (Fig. 2C, D).

As we analyzed the effect of MSC passaging on HLA-DR expression, we observed that a greater percentage of MSCs from donor 1 expanded with PL-182 expressed HLA-DR at P1 than MSCs from the same donor at P0 (*p* < 0.04; Fig. 2E). In contrast, the percentage of MSCs-P1 expressing HLA-DR from donor 2 expanded in PL-240 was lower than that of P0 (*p* < 0.005; Fig. 2F). In addition, a significantly higher percentage of MSCs from donor 3 expanded in PL-170 expressed HLA-DR at P1 than at P0 (*p* < 0.02; Fig. 2G).

According to Bocelli-Tyndall et al. [20], MSCs from 20 donors that were cultured in medium supplemented with FBS expressed HLA-DR (5–10%). By increasing the number of MSCs, HLA-DR expression declined. Moreover, these authors did not observe any increase in HLA-DR expression when MSCs were cultured in medium supplemented with PL, and as a result, they concluded that PLs do not affect the expression of HLA-DR by MSCs.

However, when cultured in the presence of bFGF, MSCs proliferated more and expressed higher levels of HLA-DR [20, 27, 28]. Although we did not specifically add bFGF in our experiments, a significant percentage of MSCs from donors 1 and 2 expressed HLA-DR, without any significant difference in proliferation capacity.

The upshot of these data is that some MSC batches are more prone than others to express HLA-DR during culture, i.e., it might be a constitutive feature. We asked whether this HLA-DR expression might be linked to the expression levels of HLA-DR in the BM-MNCs from which the MSCs were generated. Flow cytometry analysis demonstrated that BM-MNCs from 3 donors expressed HLA-DR in the range of 29–42.9%, i.e., we found no correlation between HLA-DR expression on BM-MNCs and MSCs generated thereof (data not shown).

**Fig. 3:**
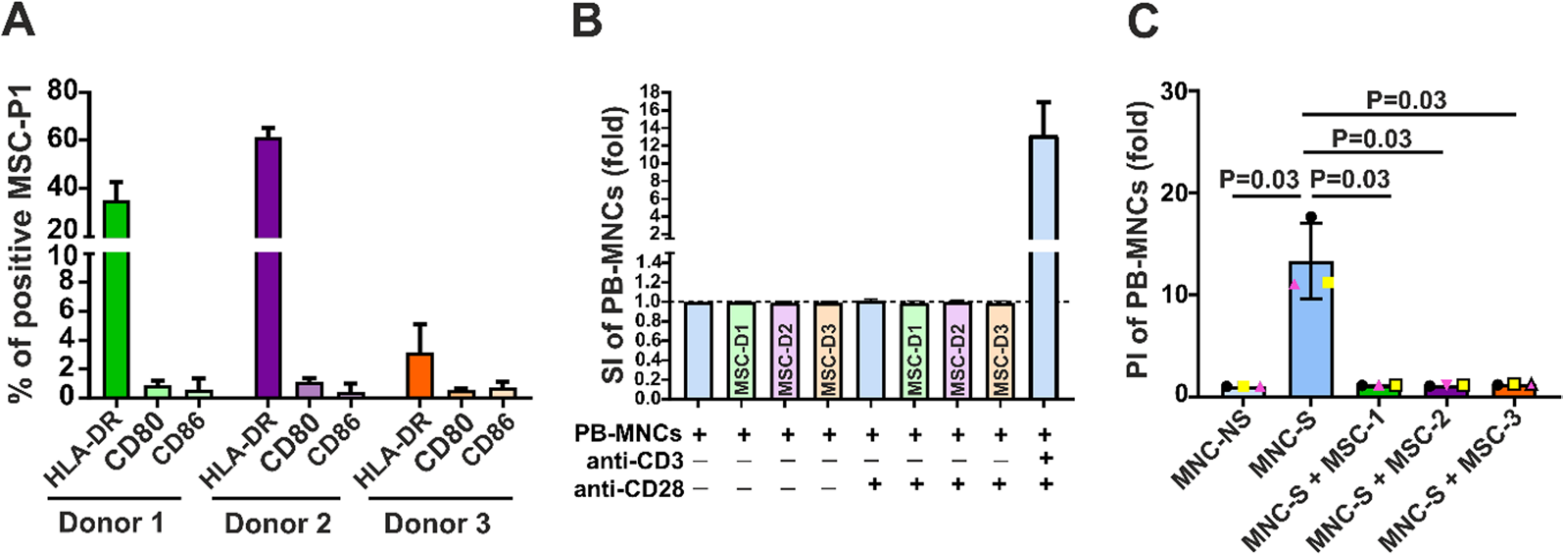
Expression of HLA-DR and costimulatory molecules as well as stimulation and proliferation index. **A** MSCs from P1 of 3 donors were expanded in 10% PL-240 for 7 days. The percentages of HLA-DR, CD80 and CD86 are presented as the mean values of 3 experiments. **B** Immunogenicity of MSCs. MSCs from 3 donors (MSC-D1, MSC-D2 and MSC-D3) were cocultured at a ratio of 1:1 with PB-MNCs isolated from buffy coats. In the other wells, MSCs were cocultured at a ratio of 1:1 with PB-MNCs isolated from buffy coats that were stimulated with anti-CD28 antibodies. PB-MNCs stimulated with anti-CD3 and anti-CD28 antibodies were used as a positive control. On day 5, the proliferation of PB-MNCs was assessed by flow cytometry, and the results are presented as the stimulation index (SI), which is expressed as the number of plated PB-MNCs (± anti-CD28) + MSCs on day 5/number of PB-MNCs (± CD28) on day 5. The results are presented as the mean ± SD of 3 experiments. There was no proliferation of either stimulated or not-stimulated MNCs with anti-CD28 antibody in the presence of MSCs. **C** Immunosuppressive effect of MSCs from 3 donors (MSC-1, MSCs-2 and MSC-3) cocultured with anti-CD3- and anti-CD28-stimulated PB-MNCs at a 1:1 ratio for 5 days. This effect is presented as the proliferation index (PI). The results were tested with a t test under the assumption of a normal distribution (N = 3). SI Index = MNCs ± anti-CD28 + MSCs/MNCs ± anti-CD28 presented in fold of proliferation. PI index = MNCs stimulated with anti-CD3/anti-CD28 (S) + MSCs/MNCs stimulated with anti-CD3/anti-CD28 (S) presented as fold changes in proliferation. MNC-S = stimulated with anti-CD3/anti-CD28; MNC-NS = not stimulated

We also asked whether this constitutively higher expression of HLA-DR might be linked to the expression of costimulatory molecules such as CD80 and CD86. Flow cytometry analysis showed low levels of these antigens, suggesting that these MSCs may not elicit an immune response (Fig. 3A).

To assess whether MSCs are immunogenic, we performed an MLR with MSCs from 3 donors. Two of them expressed high levels of HLA-DR, whereas the third donor’s MSCs expressed low levels of HLA-DR. As CD28 antigen on T cells is a costimulatory molecule that interacts with CD80 and CD86 on antigen-presenting cells, we investigated whether MSCs are able to stimulate the proliferation of PB-MNCs in the presence or absence of anti-CD28 antibodies. In the presence of MSCs:MNCs at a ratio of 1:1, we found no proliferation of PB-MNCs in either nonstimulated samples (control: SI = 0.99 ± 0.001) or anti-CD28 antibody-stimulated samples (SI: 0.98 ± 0.02) (Fig. 3B), suggesting that MSCs might not be immunogenic. In contrast, PB-MNCs stimulated with anti-CD3 and anti-CD28 demonstrated a 13-fold higher proliferation rate (PI = 13.3 ± 3.8) than nonstimulated cells (Fig. 3B). Our results showed that irrespective of HLA-DR expression levels, MSCs from all 3 donors similarly inhibited PB-MNC proliferation (p = 0.03) (Fig. 3C).

**Fig. 4:**
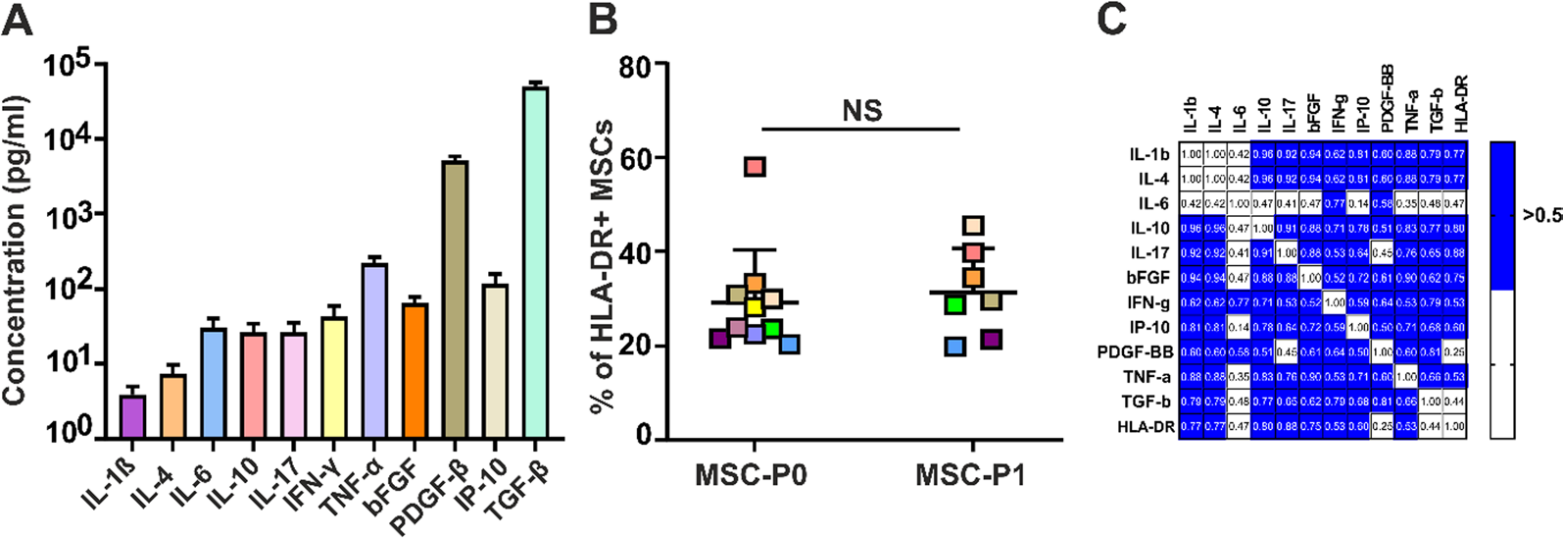
Cytokine levels in PLs and their impact on the expression of HLA-DR. **A** The levels of 11 cytokines in 10 PLs used for experiments. **B** MSCs from one donor were generated from BM-MNCs cultured in 10 different PLs. HLA-DR levels were measured by flow cytometry in P0 MSCs cultured in PL-145, PL-146 PL-166, PL-170, PL-171, PL-172 and PL-173, PL-175, PL-179 and PL-182 depicted with different colors. MSCs were expanded to P1 with the same PLs (except PL-175, PL-179 and PL-182), and HLA-DR expression of MSCs-P1 was assessed. Analysis of the results was performed by the Mann‒Whitney test (U test) because the samples did not show a normal distribution. **C** Spearman correlation analysis between the cytokine levels in PLs and expression of HLA-DR by MSCs

Because we noticed that HLA-DR expression by MSCs is more frequent when they are cultured with different batches of PL than FBS, we compared the concentrations of 11 cytokines (IL-1β, IL-4, IL-6, IL-10, IL-17, bFGF, IFN-γ, IP-10 (CXCL10), PDGF-BB, TNF-α and TGF-β) in 16 PLs and 5 batches of FBS. In Fig. 4A, the concentrations of 11 cytokines in 16 different PLs are presented, of which 10 were used to generate MSCs from the same donor presented in Fig. 4B. We found that different PLs induced a similar percentage of HLA-DR-positive MSCs in both P0 and P1 (Fig. 4B).

A significant positive correlation between the levels of cytokines in 10 PLs, which were used for the generation and expansion of MSCs, and the expression levels of HLA-DR by MSCs (Fig. 4C) was found for IL-1β (p = 0.01), IL-4 (p = 0.01), IL-10 (p = 0.007), IL-17 (p = 0.001) and bFGF (p = 0.01). In contrast, Grau-Vorster et al. [35] found no correlation of HLA-DR expression with the levels of these cytokines in human serum B used for expansion of MSCs but did find a correlation with IL-33 and IL-17F. The concentration of IL-17F was 55.94 pg/mL in human serum B, approximately twofold higher than our IL-17 levels in PLs used for the expansion of MSCs (26.1 ± 8.8 pg/mL). Notably, the levels of IL-1β, IL-10, and IL-17 in PLs used for expansion of our MSCs were similar to the concentrations of the same cytokines in PLs found in previous reports [36, 37]. The concentration of TGF-β in 10 different PLs, as presented in Fig. 4, was 50.2 ± 6.9 ng/mL, which is similar to the cytokine values reported by other groups [20, 38, 39]. The concentration of bFGF, as an important cytokine for the expression of HLA-DR on MSCs, in 10 analyzed PLs was approximately sevenfold higher (65.3 ± 13.7 pg/mL) than that in 5 batches of FBS (9.2 ± 0.5 pg/mL). Higher values of bFGF in PLs were found by other groups [20, 37–39].

**Fig. 5:**
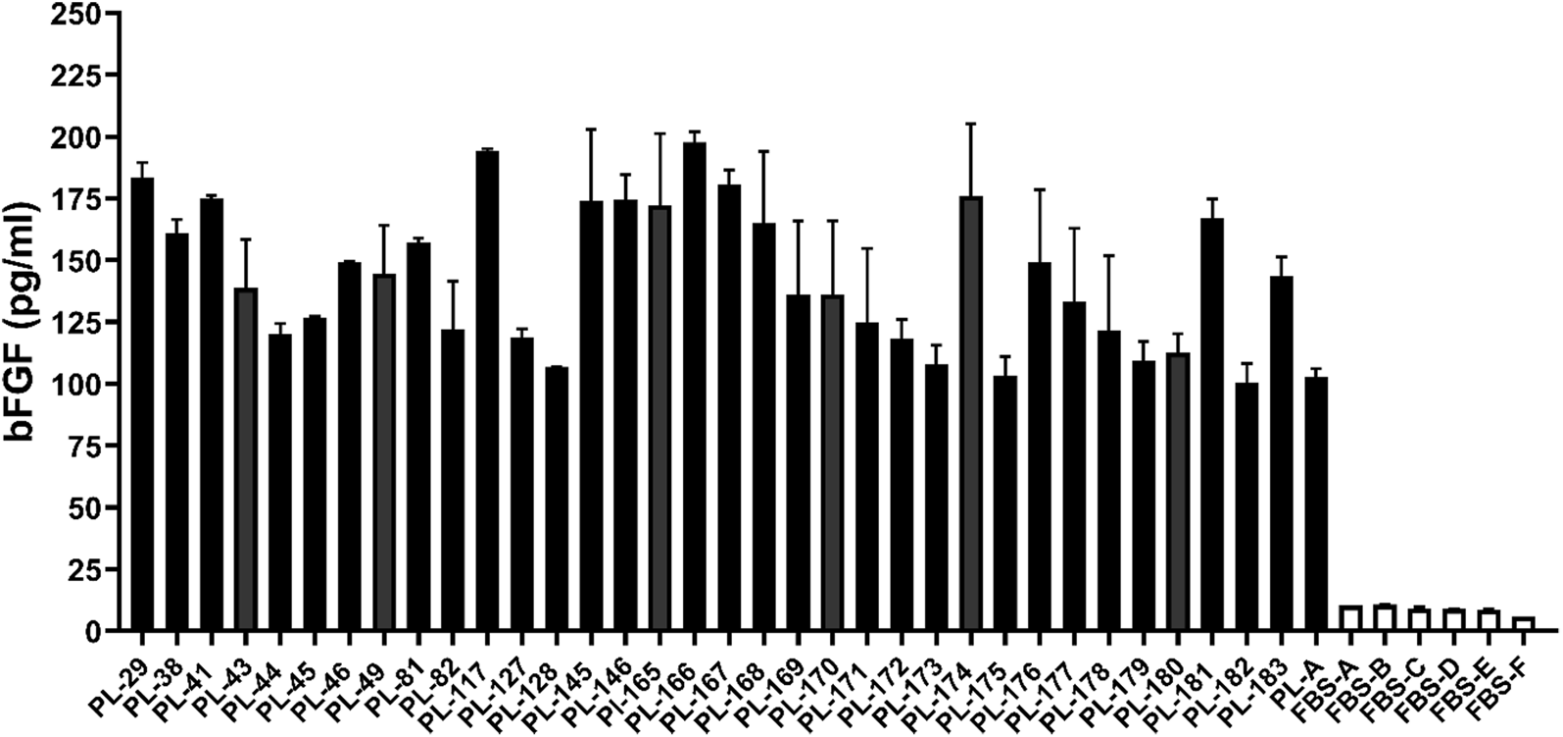
bFGF levels in platelet lysates and “MSC-qualified” FBS. This figure presents bFGF levels in 35 different batches of PLs and 6 batches of FBS. The results are presented as the mean ± SD of duplicate measurements

According to some reports, bFGF is one of several/few important factors that stimulates the proliferation of MSCs and increases HLA-DR expression. We assessed the levels of this cytokine once more in 6 different batches of FBS and 35 different PLs. (Fig. 5).

The mean bFGF concentration in FBS (8.9 ± 1.7 pg/mL) was approximately 16-fold lower than that in PLs (143.0 ± 28.9 pg/mL). Similar values of bFGF have been reported in several other studies [20, 38, 39]. We further asked whether the expression of HLA-DR can be manipulated by stimulation with TGF-β1. To investigate this phenomenon, we selected the same donor, whose MSCs in a high percentage are constitutively positive for HLA-DR (Fig. 4). MSCs were generated in 4 different PLs (PL-172, PL-173, PL-145 and PL-146) with different doses of TGF-β1 (10 ng/mL, 5 ng/mL and 2.5 ng/mL), which were exogenously added on days 0, 3, 7 and 10. On day 13, the MSCs of P0 were further passaged to P1 by being treated with the same substances on day 0 and day 4. After each passage, HLA-DR expression was evaluated. A proportion of MSCs of P1 were frozen for further RT‒PCR analysis of IDO, TGF-β1, CIITA P/S/T (CIITA upstream region) and CIITA LRR (downstream region).

RT‒PCR analysis of both nontreated and treated MSCs with TGF-β1 demonstrated that higher expression of HLA-DR was also followed by higher amounts of gene products of CIITA. TGF-β1 treatment of MSCs almost completely abrogated the expression of CIITA. Interestingly, nontreated MSCs that expressed higher levels of HLA-DR as assessed by flow cytometry after their expansion in PL-173, PL-145 and PL-146 also showed the presence of gene products for IDO in a direct proportion, i.e., the higher the level of HLA-DR antigens, the more gene products for IDO. This finding is in contrast to other reports [40–42] that generated and expanded MSCs with FBS and Chinnadurai et al. [43], who generated MSCs with PL and found that nontreated MSCs were negative for IDO. Notably, in our experiments, TGF-β1 was able to completely abrogate IDO expression. However, RT‒PCR analysis demonstrated no expression of TGF-β1 either by untreated or by MSCs treated with exogenous TGF-β1, in contrast to Ryan et al. [41] and Yoo et al. [40], who generated MSCs with FBS and found that untreated MSCs expressed TGF-β1.

Next, we stimulated MSCs generated from another donor with 500 UI/mL IFN-γ for 24 and 48 h to test its effect on the expression of TGF-β1. Both untreated MSCs and MSCs treated with IFN-γ for 24 h did not express TGF-β1. Only MSCs treated with IFN-γ for 48 h were weakly positive for TGF-β1 in RT‒PCR, in contrast to significantly increased levels of CIITA and HLA-DR (Table 2).

**Table 2:** PCR analysis of MSCs in P1 for IDO, CIITA, TGF-β1 and HLA-DR (flow cytometry)

MSC-181116^**a**^	2,3-IDO ng/µL	CIITA ng/µL	TGF-β1 ng/µL	HLA-DR (%)
MSCs-P1/PL-269	^b^ND	52.1	ND	23.4
MSCs-P1/PL269 + IFN-γ 24 h	39.1	63.9	ND	95.0
MSCs-P1/PL269 + IFN-γ 48 h	34.5	54.6	1.7	95.0

^a^MSCs of donor 181,116 after generation from BM-MNCs at P0 expressed high levels of HLA-DR, P0 = 31%

^b^ND = Not Detected

**Fig. 6:**
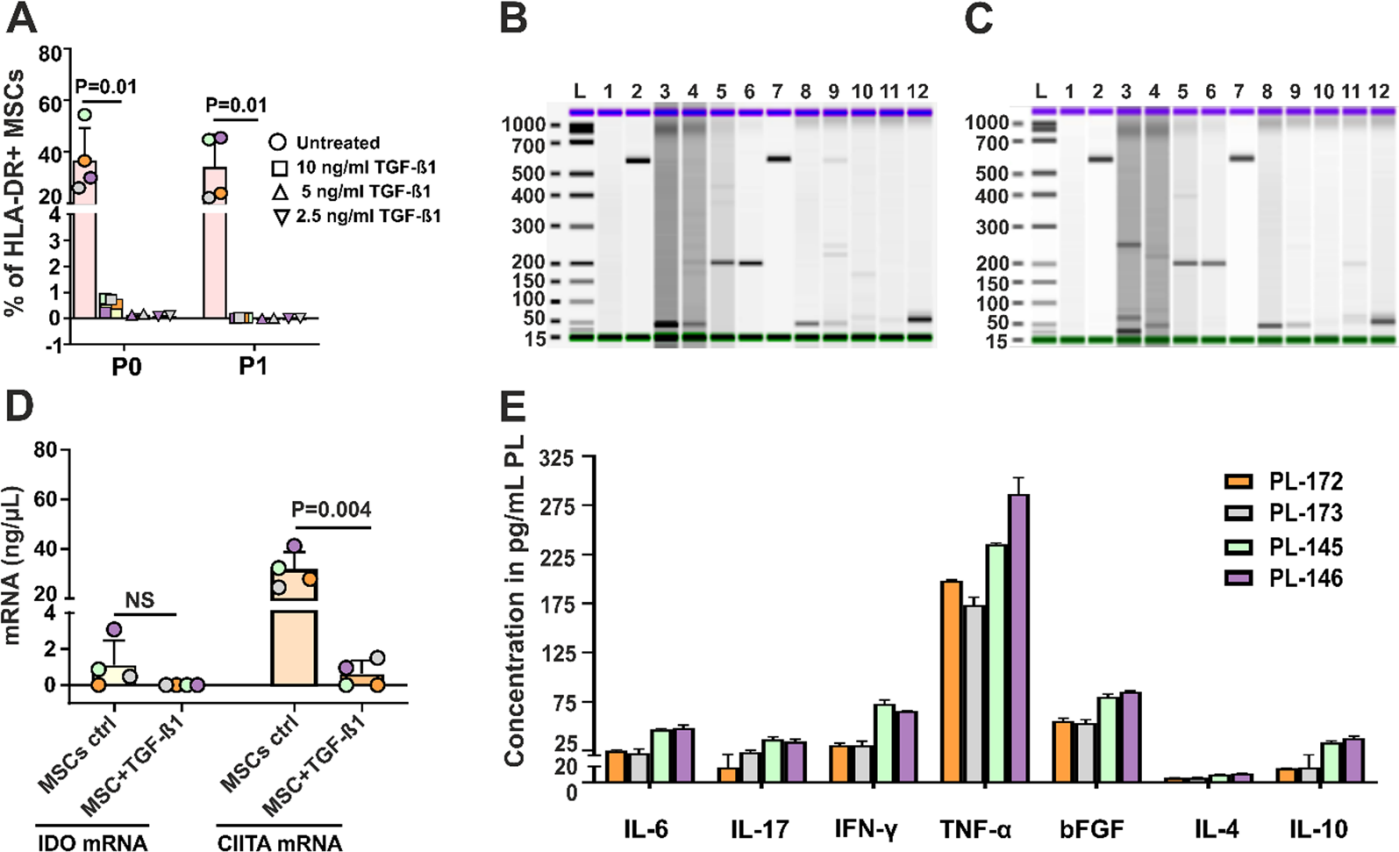
The effect of TGF-β1 on HLA-DR expression by MSCs and RT‒PCR analysis of CIITA, IDO and TGF-β1. **A** The effect of exogenously added TGF-β1 at repetitive doses on the expression of HLA-DR by MSCs in P0- and P1-cultured PL-145 (green circles), PL-146 (purple circles), PL-172 (orange circles) and PL-173 (gray circles) cells as measured by flow cytometry. Values represent the mean ± SD (paired t test) of 4 independent experiments with 4 different PLs. **B** RT‒PCR analysis of MSCs in P1 after repetitive treatment with TGF-β1 from P0 to P1. RT‒PCR of P1 MSCs generated in PL-172 cells. Lane L: loading marker, Lane 1: H_2_O, Lane 2 to Lane 6: MSCs untreated, control: Lane 2: GAPDH, Lane 3: IDO, Lane 4: TGF-β1, Lane 5: CIITA (upstream region), Lane 6: CIITA (downstream region), Lane 7 to Lane 11: MSCs treated with TGF-β1, Lane 7: GAPDH, Lane 8: IDO, Lane 9: TGF-β1, Lane 10: CIITA (upstream region), Lane 11: CIITA (downstream region). Lane 12: control (water instead of cDNA in PCR). **C** RT‒PCR products of MSCs in P1 after repetitive treatment with TGF-β1 from P0 to P1. MSCs-P1 were generated in PL-146. Designation of lines is the same as in (**B**).** D** RT‒PCR products (mRNAs) for IDO and CIITA in MSCs cultured with PL-145 (green circles), PL-146 (purple circles), PL-172 (orange circles) and PL-173 (gray circles) ± TGF-β1. **E** Concentration of cytokines in platelet lysates that were used for culture of MSCs in all experiments presented in this figure

Because of the positive correlation of RT‒PCR results with the data generated by flow cytometry, we analyzed the levels of cytokines in PLs that were used for expansion of MSCs in Fig. 6A. It is apparent from Fig. 6E that lysates that induce a higher expression of HLA-DR in MSCs contain more inflammatory cytokines (IL-6, IL-17, IFN-γ, TNF-α) as well as bFGF. In addition to changes in the expression of HLA-DR by cultured MSCs in different PLs (Fig. 6A), we also assessed mRNA levels for IDO and CIITA in these cells (Fig. 6D). Flow cytometric analysis of MSCs generated from the same bone marrow donor in 4 different PLs (PL-172, PL-173, PL-145 and PL-146) demonstrated varying levels of HLA-DR expression between 24.0% and 45.5%. RT‒PCR demonstrated that nonlicensed MSCs also expressed some CIITA, but MSCs that expressed higher levels of HLA-DR also showed higher amounts of CIITA transcripts. Other authors reported no expression of CIITA by MSCs generated in FBS [20], in contrast to Tang et al. [19], who demonstrated a slight expression of CIITA in MSCs that were generated in FBS from whole bone marrow.

Interestingly, TGF-β1 potently suppressed HLA-DR expression by MSCs in culture, as determined by flow cytometry. This was consistent with the results of RT‒PCR in which no expression of CIITA in two PLs or a slight expression of these transcripts in two other PLs was found. In addition, we also assessed the levels of mRNA for IDO and found that untreated MSCs, which were generated and expanded in 3 different PLs (PL-173, PL-145 or PL-146), expressed high levels of HLA-DR (22.2%, 44.8% and 45.5%, respectively) and IDO transcripts (Fig. 6D). However, only MSCs generated with PL-172 (Fig. 6D) that expressed HLA-DR (24.0%) in RT‒PCR expressed no IDO transcripts. Moreover, MSCs expanded in 4 different PLs expressed no TGF-β1 transcript in RT‒PCR, in contrast to other authors that showed that MSCs were negative for IDO but positive for TGF-β1 [40, 41]. Notably, the MSCs in these reports were generated and expanded in FBS.

Because nontreated MSCs with high levels of HLA-DR also express IDO, we treated MSCs from three other donors at P2 with TGF-β1 (10 ng/mL) alone, IFN-γ (500 UI/mL) alone and TGF-β1 (10 ng/mL) + IFN-γ (500 IU/mL) together 24 h before trypsinization. For this purpose, MSCs from two donors (MSC-18116 and MSC-220922) were used, which constitutively expressed high levels of HLA-DR at P0 (31.0% and 34.9%, respectively), whereas MSCs from the other donor (MSC-310821) at P0 expressed less HLA-DR (3.7%) (Table 3).

**Table 3:** Gene expression of IDO, CIITA and HLA-DR in MSCs of P2

MSC donors	2,3-IDO ng/µL	CIITA ng/µL	HLA-DR %
MSCs-310821-P2/PL-240	ND	46.9	6.9
MSCs-310821-P2/PL-240 + TGF-β1	ND	17.1	2.7
MSCs-310821-P2/PL-240 + IFN-γ	50.9	95.6	58.3
MSCs-310821-P2/PL-240 + IFN-γ + TGF-β1	35.1	67.3	40.1
MSCs-181116-P2/PL-269	ND	55.8	^a^6.3
MSCs-181116-P2/PL-269 + TGF-β1	ND	16.1	4.9
MSCs-181116-P2/PL-269 + IFN-γ	42.6	74.8	95.4
MSCs-181116-P2/PL-269 + IFN-γ + TGF-β1	36.9	64.5	93.4
MSCs-220922-P2/PL-269	1.4	55.2	29.5
MSCs-220922-P2/PL-269 + TGF-β1	ND	36.8	33.6
MSCs-220922-P2/PL-269 + IFN-γ	52.0	76.9	91.2
MSCs-220922-P2/PL-269 + IFN-γ + TGF-β1	42.9	62.5	90.3

^a^MSCs of donor 181,116 after generation from BM-MNCs at P0 expressed high levels of HLA-DR, P0 = 31%

ND = not detected

RT‒PCR analysis showed that MSCs expressing high levels of HLA-DR (MSCs-220922) also expressed IDO. To the best of our knowledge, this is the first report that MSCs cultured in PL constitutively express IDO, even though in significantly lower amounts than after treatment of MSCs with IFN-γ. Treatment of MSCs for 24 h with TGF-β1 before trypsinization induced a reduction in constitutive HLA-DR expression, in contrast to treatment with IFN-γ and TGF-β1, in which the HLA-DR expression levels were almost unaffected. On the other hand, treatment of MSCs with TGF-β1 induced a complete abrogation of constitutive expression of IDO. Only a small reduction in its expression was induced when MSCs were licensed with IFN-γ. This high HLA-DR expression may be partly explained by the fact that these PLs showed a higher concentration of IFN-γ (88-fold) and bFGF (tenfold) than FBS (data not shown). In turn, the concentration of TGF-β1 was only fivefold higher than that of FBS, indicating that this cytokine could not effectively inhibit HLA-DR expression by MSCs. Irrespective of their HLA-DR expression, MSCs effectively suppressed the proliferation of PB-MNCs stimulated with anti-CD3/CD28 in MLRs. MSCs themselves were unable to induce proliferation of not stimulated PB-MNCs from an HLA-disparate donor. This suggested that these MSCs are not able to play the role of antigen-presenting cells, because they lack expression of costimulatory molecules (CD80 and CD86).

## Conclusions

In the current study, we demonstrate that some primary cultures of platelet lysate-expanded MSCs express HLA-DR. While primarily a property of the individual MSC batch, some variation was due to the platelet lysate batch. The expression of HLA-DR affected neither the proliferative nor allosuppressive potential of MSCs. There was a positive correlation between the concentration of 5 out of 11 tested cytokines in 10 PLs and HLA-DR expression. All tested MSCs generated with PLs expressed CIITA. Most MSCs generated with PLs that constitutively expressed CIITA and a high percentage of HLA-DR in P0 or P1 also expressed moderate levels of transcripts for IDO, an association that had previously not been reported. Neither MSCs, which expressed high levels of HLA-DR, nor MSCs with low levels of HLA-DR were able to elicit an in vitro immune response. In addition, RT‒PCR analysis demonstrated that HLA-DR-positive MSCs showed a positive correlation between the expression of CIITA and IDO.

Taken together, our results provide and support the statement that HLA-DR expression by MSCs does not correlate with any biologically or pharmacologically relevant function. We consider HLA-DR to be an obsolete release marker for the clinical application of MSCs and therefore propose omitting it from the defining surface antigen panel of *bona fide* MSCs.

## Data Availability

The original contributions presented in the study are included in the article. Further inquiries can be directed to the corresponding author.

## Abbreviations

ISCT: International Society for Cell and Gene Therapy
MSCs: Mesenchymal stromal cells
PLs: Platelet lysates
FBS: Fetal bovine serum
IFN-γ: Interferon-gamma
TNF-α: Tumor necrosis factor alpha
TGF-β: Transforming growth factor beta
CIITA: Class II Transactivator
IDO: Indoleamine 2,3-dioxygenase
MLR: Mixed Lymphocyte Reaction
PB-MNCs: Peripheral blood mononuclear cells
BM-MNCs: Bone marrow mononuclear cells
RT‒PCR: Real-time Polymerase Chain Reaction
FACS: Fluorescent Activated Cell Sorting
